# Metabolic Syndrome as a Factor of Impairment of Antioxidant Defense System in Youth with T1DM

**DOI:** 10.3390/ijms24119428

**Published:** 2023-05-29

**Authors:** Monika Grabia, Katarzyna Socha, Artur Bossowski, Renata Markiewicz-Żukowska

**Affiliations:** 1 Department of Bromatology, Faculty of Pharmacy with the Division of Laboratory Medicine, Medical University of Białystok, Mickiewicza 2D Street, 15-222 Białystok, Poland; monika.grabia.diet@gmail.com (M.G.);; 2 Clinic of Pediatrics, Endocrinology, Diabetology with the Subdivision of Cardiology, Children’s University Clinical Hospital in Białystok, Waszyngtona 17 Street, 15-274 Białystok, Poland

**Keywords:** adolescents, diabetes mellitus type 1, metabolic syndrome, antioxidant enzymes, antioxidant status, dyslipidemia, hypertension, obesity, continuous glucose monitoring, biomarkers

## Abstract

Research indicates that adolescents with type 1 diabetes mellitus (T1DM) may develop both metabolic syndrome (MetS) and oxidative stress. The purpose of this study was to test the hypothesis that MetS could potentially affect antioxidant defense parameters. The study recruited adolescents aged 10–17 who had been diagnosed with T1DM, and divided them into two groups: “MetS+” (n = 22), who had been diagnosed with MetS, and “MetS−” (n = 81), who did not have metabolic syndrome. A control group consisting of 60 healthy peers without T1DM was included for comparison. The study examined cardiovascular parameters, such as complete lipid profile and estimated glucose disposal rate (eGDR), as well as markers of antioxidant defense. The results revealed a statistically significant difference between the MetS+ and the MetS− group in terms of total antioxidant status (TAS) (1.186 mmol/L vs. 1.330 mmol/L), and oxidative stress index (OSI) levels (0.666 vs. 0.533). Furthermore, multivariate correspondence analysis identified individuals with HbA1c < 8%; eGDR > 8 mg/kg/min, using either flash or continuous glucose monitoring systems, as MetS− patients. The study also found that eGDR (AUC 0.85, *p* < 0.001), OSI and HbA1c (AUC 0.71, *p* < 0.001) markers may be useful for diagnosing the onset of MetS in adolescents with T1DM.

## 1. Introduction

The most commonly diagnosed type of diabetes mellitus (DM) among children and adolescents is type 1 (T1DM), which is an autoimmune disease. There is a reduction in insulin production due to the degradation of pancreatic islet β cells, making it necessary for such patients to be treated with intensive insulin therapy for the rest of their lives [1,2,3]. Recent years of research have shown that an increasing number of adolescents are overweight [4]. It is also observed that the pediatric diabetic population is beginning to exhibit a similar trend of being overweight [5]. Most importantly, research suggests that the issue of concern is not limited to overweight or obesity, but also encompasses metabolic syndrome (MetS), which is beginning to emerge [6]. MetS is a set of factors whose presence reflects an increased risk of cardiometabolic complications [7]. Our previous study indicated that approximately 30% of patients with T1DM had MetS. Those individuals had excessive visceral fat, consumed low amounts of monounsaturated fatty acids and high quantities saturated fatty acids. What is more concerning, they also had low total antioxidant status (TAS) and poorly controlled T1DM [8]. This is particularly important because maintaining proper glycemic control is essential in preventing the disorders associated with the antioxidant defense system (AOD) through the accumulation of reactive oxygen species (ROS). Considering previous findings, it is possible that oxidative stress (OS) is a factor which could contribute to the accelerated progression of diabetes complications [8,9].

The purpose of this study was to investigate the presence of oxidative stress in adolescents with T1DM who developed MetS.

## 2. Results

Table 1 presents basic information that characterizes the study participants based on their MetS diagnosis. The data showed that 21% of diabetic participants met the diagnostic criteria for MetS: they had a statistically significantly higher (*p* < 0.05) body weight compared to those in the MetS− group. Additionally, there was a higher prevalence of MetS among adolescents with a T1DM duration exceeding 2 years (*p* < 0.001). Moreover, it was more common among individuals who solely relied on a glucometer instead of modern glucose monitoring systems (GMS) (*p* < 0.001) and those using CSII (*p* < 0.001).

The study findings indicated that there were significant differences in all cardiovascular markers between the MetS+ and MetS− groups, as well as between the MetS+ group and healthy peers, except for low-density lipoprotein (LDL) (Table 2). It was observed that MetS+ diabetic patients had statistically significantly lower TAS (MetS+ vs. MetS−: 1.186 mmol/L vs. 1.330 mmol/L, *p* < 0.05) and higher levels of oxidative stress index (OSI) (0.666 vs. 0.533, respectively, *p* < 0.01). No statistically significant differences were observed between boys and girls in MetS+, as well as MetS− group for all the parameters included in this table. 

**Figure 1:**
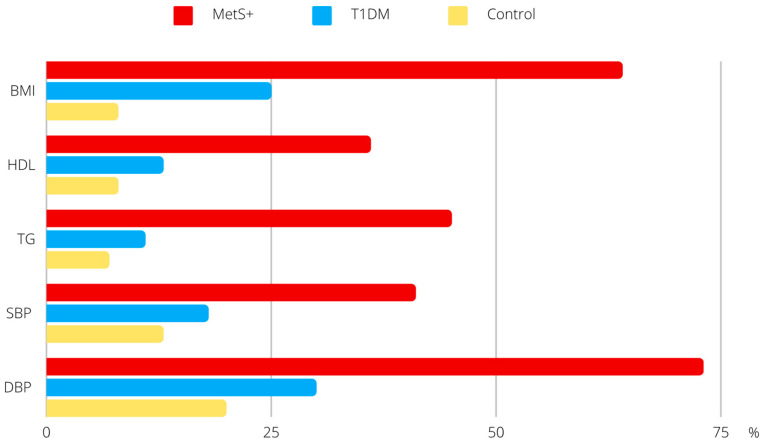
Percentage of participants meeting the components of MetS. Values are expressed as percentage of respondents (%). Abbreviations: body mass index (BMI), diastolic blood pressure (DBP), high-density lipoprotein (HDL), metabolic syndrome (MetS), systolic blood pressure (SBP), diabetes mellitus type 1 (T1DM), triglycerides (TG).

**Figure 2:**
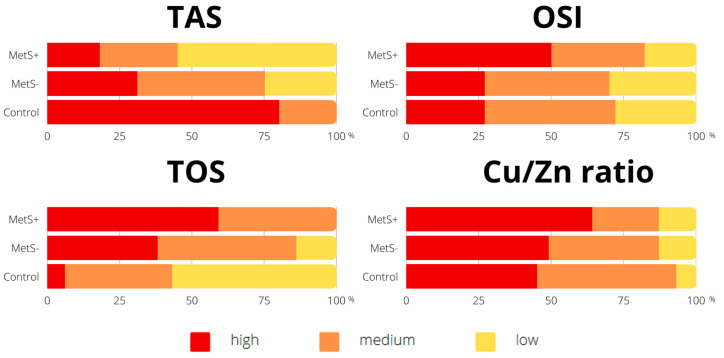
Percentage of participants according to the intensity of total antioxidant status and oxidative stress parameters. Values are expressed as percentage of respondents (%). Abbreviations: copper (Cu), metabolic syndrome (MetS), oxidative stress index (OSI), total antioxidant status (TAS), total oxidant status (TOS), zinc (Zn).

The two most prevalent components of MetS were high diastolic blood pressure (DBP) and a body mass index (BMI) (Figure 1). In addition, statistically significant relationships were found between the intensity of antioxidant defense, oxidative stress parameters, and the presence of MetS (Figure 2). Compared to participants without MetS, the majority of MetS+ individuals had lower TAS levels (MetS+ vs. MetS−: 55% vs. 25%, *p* < 0.001), higher total oxidant status (TOS) (59% vs. 38%, *p* < 0.01), OSI (50% vs. 27%, *p* < 0.001), and a higher Cu/Zn ratio (64% vs. 49%, *p* < 0.05).

Table 3 displays statistically significant correlations among selected studied parameters in diabetics with MetS. Notably, there were high statistically significant correlations between glycated hemoglobin (HbA1c) and estimated glucose-disposal rate (eGDR) (R = −0.8, *p* < 0.001), triglycerides (TG) (R = 0.6, *p* < 0.01), systolic blood pressure (SBP) (R = 0.5, *p* < 0.05), and TAS (R = −0.5, *p* < 0.05). Additionally, as DBP increased, there was a decrease in eGDR (R = −0.7, *p* < 0.001), and an increase in BMI (R = 0.6, *p* < 0.001).

**Figure 3:**
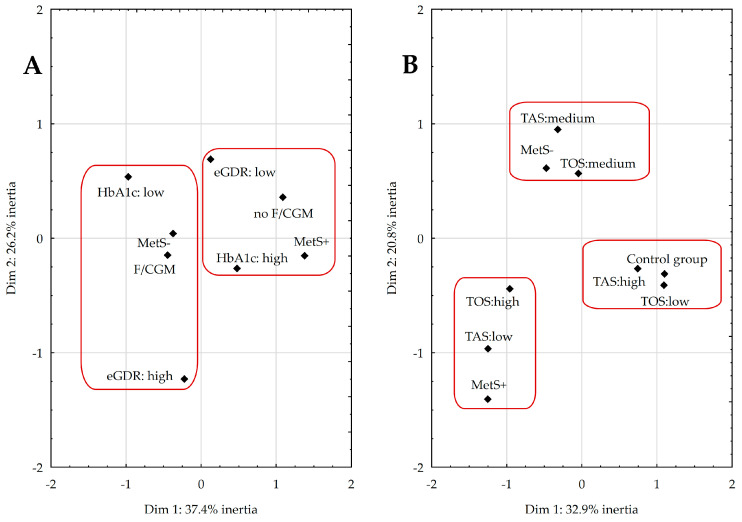
Coordinate plot for the multivariate correspondence analysis illustrating the relationship between metabolic management and the use of modern glucose monitoring systems (**A**) and the body’s antioxidant–oxidant balance (**B**). Abbreviations: continuous glucose monitoring (CGM), estimated glucose-disposal rate (eGDR), flash glucose monitoring (FGM), glycated hemoglobin (HbA1c), metabolic syndrome (MetS), total antioxidant status (TAS), total oxidant status (TOS).

Multivariate correspondence analysis (MCA) was performed to investigate the relationships between the presence of MetS, metabolic management, and the use of modern GMS (Figure 3A), as well as the link between MetS and the body’s AOD system (Figure 3B). 

In Figure 3A:

(1) The first and fourth quadrants include MetS+ participants with poor metabolic management (HbA1c ≥ 8% and eGDR ≤ 8 mg/kg/min), using only glucometers without any modern GMS.
(2) The two left quadrants, II and III, consist of diabetic patients without MetS using either flash glucose monitoring (FGM) or continuous glucose monitoring (CGM), with better metabolic management (HbA1c < 8% and eGDR > 8 mg/kg/min).

Together, these quadrants accounted for 64% of the total variability in the data.

In Figure 3B:

(1) The second quadrant consists of diabetics without MetS characterized by moderate levels of TAS and TOS.
(2) The third quadrant contains individuals from the MetS+ group with low TAS and high TOS.
(3) The fourth quadrant (IV) consists of healthy peers with high TAS and low TOS.

Together, these quadrants accounted for 54% of the total variability in the data.

Table 4 presents the results of ROC analysis, which showed significant diagnostic value for eGDR (AUC 0.85, *p* < 0.001), OSI and HbA1c (AUC 0.71, *p* < 0.001), TAS (AUC 0.67, *p* < 0.01), and TOS (AUC 0.63, *p* < 0.05). The cutoff point for HbA1c identified 73% of those with MetS and 30% of those without MetS. 

## 3. Discussion

Our study confirmed the hypothesis that diabetics with MetS experienced AOD impairment. In addition to observing low TAS and high OSI levels in this group of patients, we also demonstrated that improvements in their condition could be achieved through better metabolic control of the disease (HbA1c < 8%; eGDR > 8 mg/kg/min) and implementation of one of the F/CGM systems. These systems are becoming increasingly widespread, not only improving the quality of life of patients, but also helping maintain appropriate glycemic control, which is a key element of preventive OS [10,11].

Obesity is characterized by an excessive accumulation of body fat, especially visceral adipose tissue. Unfortunately, it continues to rise in the population and is a major contributor to the development of hypertension, or dyslipidemia, thereby increasing the risk of MetS [12]. Its progression, combined with the ongoing advancement of T1DM, has the potential to promote oxidative stress, which was found to be present among the examined patients in the current study. We have demonstrated that MetS+ patients had an impaired antioxidant defense system (Table 2), as evidenced by statistically significantly lower TAS and higher TOS levels, as well as low activity of superoxide dismutase (SOD) and catalase (CAT) when compared to the control group. In addition, among MetS+ patients, 73% had elevated DBP, and 64% had high BMI (Figure 1). Studies have suggested that ROS influence the regulation of endothelial function and vascular remodeling of their production, which may contribute to the development of hypertension [13]. Furthermore, excessive body weight is strongly associated with adipocyte dysfunction and the secretion of pro-inflammatory adipokines, which can lead to a depletion of AOD system reserves [8,14]. Among T1DM patients, especially those who are metabolically imbalanced, this process seems to be more compounded. The increase in HbA1c levels in our study participants was accompanied by a decrease in TAS (Table 3) which, according to other studies, further exacerbates the degradation of pancreatic islet β cells [15]. To prevent complications in T1DM, it is crucial to maintain normoglycemia. The presence of hyperglycemia, even for a short time, can lead to a phenomenon called “metabolic memory”. This phenomenon induces irreversible changes in cellular function, involving the advanced glycation end products receptor and other factors, leading to a cascade of events associated with inflammation and OS progression. Despite subsequent return to an optimal state, these changes can persist and contribute to long-term complications [16]. In our study, we also observed that an increase in HbA1c marker was accompanied by a decline in eGDR and an increase in TG and SBP (Table 3).

Diabetes and obesity both promote glycoxidation, leading to enzymatic changes in the body by causing cell dysfunction, resulting in disruption of the AOD system [17]. In the MetS patients that we studied, both conditions occurred together, making it difficult to determine exactly which pathway is activated by which factor. Obesity and OS are closely related through maintenance mechanisms. OS can trigger obesity and also be a consequence of it. Nutritional factors such as excessive eating, or high-fat as well as high-carbohydrate diets, can activate intracellular pathways, such as NOX, oxidative phosphorylation in mitochondria, or glycoxidation, thereby enhancing OS [17,18]. In the development of metabolic disorders, the proliferation of preadipocytes due to chronic adipocyte inflammation, fatty acid oxidation, or accumulation of cellular damage can trigger ROS. Furthermore, adiponectin promotes high LDL and low HDL concentrations and shows a negative association with BMI, but a positive association with pro-inflammatory cytokines, such as TNF-α and IL-6 [19,20,21]. The lipid profile results obtained in our study were similar to those reported by other Authors who also studied young individuals with T1DM [10,22,23].

One straightforward parameter to calculate as an indicator of insulin resistance (IR) is the eGDR (Table 2). It was significantly lower (4.4 mg/kg/min) in the MetS+ group in our study, compared to the result obtained by Köken et al. in a similar group (6.4 mg/kg/min) [24]. Köken et al. also suggested that eGDR could be a potential predictor for MetS in T1DM. We confirmed statistically significant diagnostic value of eGDR (Table 4). The presence of IR is associated with OS. To respond to insulin, the cell increases the expression of its main glucose transporter (GLUT 4), causing increased glucose uptake from the bloodstream. However, when insulin concentrations are excessively high, expression decreases, resulting in elevated blood glucose levels, so the pancreas continues to secrete insulin. The consequence is a deterioration of tissue sensitivity towards the hormone, leading to the development of hyperglycemia, hyperinsulinemia [25,26]. Such a condition in the body results in the activation of pathways that trigger stress transduction and increased glucose metabolism, which affects the appearance of ROS, resulting in the occurrence of intracellular OS [27]. In reaction to ROS, the organism inhibits phosphorylation of the tyrosine pathway and then blocks GLUT 4 translocation, all of which leads to a vicious cycle because it impairs glucose disposal and increases insulin secretion [25,26].

The most significant enzymes of the AOD system are SOD, CAT, and glutathione peroxidase (GPx). SOD is involved in removing superoxide ions and converting them into oxygen and less reactive hydrogen peroxides. Studies have shown that SOD may positively impact the implications of the production of ROS induced by hyperglycemia. Catalase, on the other hand, protects cells from the harmful effects of H_2_O_2_ by participating in its conversion to oxygen and water. Meanwhile, GPx is one of the primary enzymes that prevent the aggregation of intracellular H_2_O_2_ [28]. Based on the above findings, we detected high CAT activity and low SOD and GPx activity in diabetics with MetS when compared to the control group (Table 2). Similar observations were reported in other studies where the group consisted of diabetics with cardiovascular complications: a decrease in SOD and GPx was observed [29]. Furthermore, our ROC analysis (Table 4) also revealed that TAS (cutoff point 1.213 mmol/L), TOS (cutoff point 6.973 μmol H_2_O_2_ equiv./L), and OSI (cutoff point 0.575) could potentially serve as predictors of MetS and be used as one of the components.

Studies have reported that MetS can be prevented through reprogramming, even before the onset of symptoms. The suggested way to achieve this is by supporting impaired antioxidant defenses through antioxidant therapies that target various mechanisms, including enzymatic and non-enzymatic ones. The first approach offers the potential therapeutic effect of targeting individual components of MetS with SOD mimetics [30]. However, most of these studies have been conducted on animals and have relied primarily on supplementation, which makes it difficult to rank them in drug form. Therefore, evidence from ongoing clinical trials is necessary to validate their effectiveness [31]. Strategies involving vitamins and polyphenols, such as resveratrol, genistein, and curcumin have also been suggested as non-enzymatic mechanisms for reprogramming. However, more research is required in this case, taking into account the complexity and inter-individual variability of their pharmacokinetics, as well as their low bioavailability in vivo [30,32].

Despite the valuable insights provided by this study, as with any research, there are strengths and limitations to consider. Among the limitations is the fact that, due to the study’s design, it is not possible to establish causal relationships and measure the incidence of MetS directly. At this stage the study can only identify the presence of the problem. Nevertheless, it should be noted that our research addresses a gap in the scientific understanding, which has been repeatedly discussed in previous studies. Additionally research in this area among T1DM adolescents rarely involves a control group, which enhances the significance of our findings.

## 4. Materials and Methods

### 4.1. Study Group

The case-control study consisted of 163 participants aged 10–17 years. The T1DM group included 103 patients, of which 22 were diagnosed with MetS, while the remaining 81 did not have the condition. The diabetics were recruited between March 2020 and September 2022 from the Department of Pediatrics, Endocrinology, Diabetology with the Division of Cardiology at the University Children’s Clinical Hospital in Bialystok. Only those who met the following criteria were eligible for participation: age between 10 and 17 years, presence of T1DM, absence of other types of DM and severe chronic diseases, as well as interest in participating in the study. The diagnosis of T1DM was based on the presence of autoantibodies, as determined by physicians specializing in diabetology [1]. The control group consisted of 60 healthy volunteers without a diagnosis of MetS who visited the Department of Bromatology at the Medical University of Bialystok. At the time of recruitment, they reported an absence of any history of symptoms suggesting the presence of DM or other chronic diseases. The permission of the Bioethics Committee (No. R-I-002/587/2019) had been obtained prior to the study, and the consent of each participant’s guardian was required during the study.

### 4.2. Blood Samples Analysis 

For the collection of fasting blood as material for analysis, Vacutainer tubes containing clot activator and gel or anticoagulant K2EDTA (Becton Dickinson, Pont de Claix, France) were used. After centrifuging the blood for 10 min at about 2000 rpm (Centrifuge M-diagnostic, MPW, Warsaw, Poland), the serum was moved into tubes and stored at −20 °C to be used for the determination of mineral elements and −80 °C to provide suitable conditions for subsequent measurement of antioxidant defense and oxidative stress parameters. To determine Cu and Zn contents, the serum was first deproteinized with 1 mol/L nitric acid (Suprapur, Merck, Darmstadt, Germany), and surfactant 1% Triton X-100 (Sigma-Aldrich, St. Louis, MO, USA) was added. The samples were then centrifuged for 10 min at about 6000 rpm (Centrifuge IKA mini G, IKA, Staufen im Breisgau, Germany). Zn was measured in the supernatant, and Cu was measured in a sample that had been additionally diluted with 0.1 mol/L nitric acid. Directly prior to the determination of Se, the material was diluted with 0.2% Triton X-100. Calibration curves were performed on standard solutions (Merck, Darmstadt, Germany). The study employed the Zeeman background-corrected atomic absorption spectrometry method (Z-2000, Hitachi, Tokyo, Japan) with an acetylene-air flame atomization technique for Zn, and a flameless technique with electrothermal atomization in a graphite cuvette for Cu and Se to measure mineral concentrations. In addition, the molar ratio between Cu and Zn was calculated. To determine oxidative stress parameters, a spectrophotometry technique was applied using a microplate reader (Infinite M200 Pro Tecan, Männedorf, Switzerland). The TOS measurement was conducted following the methodology proposed by Erel et al. [33]. TAS and GPx were assayed using Randox reagent kits (Randox Laboratories, Crumlin, County Antrim, UK). The OSI was determined as the ratio of TOS to TAS. CAT and SOD were detected via reagent sets from CaymanChem (Cayman Chemical Company, Ann Arbor, MI, USA). Table 5 provides detailed information about the methods employed in the study. The limits of detection for Cu, Se, and Zn were 0.0005 mg/L, 1.44 µg/L, and 0.02 mg/L, respectively. To facilitate the interpretation of the results, categories were established for each parameter, with “medium” representing the reference range and “low” and “high” indicating values outside this range. The reference range for TAS was 1.2–1.45 mmol/L, for TOS it was 5–8 μmol H_2_O_2_ equiv./L, for OSI it was 0.3–0.6, and for the Cu/Zn ratio it was 0.6–1.0. The levels of total cholesterol (TC), TG, HDL, LDL and fasting glucose level were determined using the enzymatic colorimetric method on an Alinity c analyzer (Abbott Laboratories, Lake Bluff, IL, USA). To assess metabolic management, HbA1c was measured, and the eGDR was calculated, by means of ion-exchange high-performance liquid chromatography using a Bio-Rad D-10TM (Bio-Rad, Hercules, CA, USA). The calculation of eGDR was based on a formula that considered the value of BMI [34]. The decrease in this index reflects the increase in IR in the body. The accuracy of the methods was ensured by performing determinations with dedicated certified materials (Seronorm Trace Elements Serum L-1, Sero AS, Norway; Quality Control Randox; Catalase Control CaymanChem). 

### 4.3. Anthropometric Measurements

To perform an initial assessment of nutritional status, anthropometric measurements of body height and weight were obtained. Body height was measured in the Frankfort horizontal position, using a height meter with an accuracy of 0.1 cm. Body weight was taken using a calibrated medical instrument with an accuracy of 0.1 kg. Additionally, the body mass index was calculated using the following formula: body weight in kg divided by height in meters squared, which was referenced to national centile grids [35].

### 4.4. Metabolic Syndrome Diagnosis

The criteria for diagnosing MetS in each patient were based on those proposed in a previous publication. These criteria were modified based on other established ones, and the rationale for their use was extensively described in previous papers [6,8]. The MetS+ group included participants who met three out of the five components:

(1) BMI ≥ 95th percentile (based on Polish percentile grids [35]);
(2) TG ≥ 130 mg/dL (based on the norm for the pediatric population [36]);
(3) HDL ≤ 40 mg/dL (based on the norm for the pediatric population [36]);
(4) SBP/DBP ≥ 95th percentile (based on Polish percentile grids [37]);
(5) FGL ≥ 100 mg/dL or known DM.

### 4.5. Statistical Analysis

Statistica software (version 13 PL; TIBCO Software Inc., Palo Alto, CA, USA) was used for appropriate statistical processing of the data. To select adequate tests, normality of the distribution of variables was determined using the Shapiro–Wilk, Kolmogorov–Smirnov and Lilliefors tests. Kruskal–Wallis ANOVA test with post-hoc analysis was used to assess the significance of quantitative variables, and the chi-squared test of independence was applied to examine the relationships between qualitative variables. Spearman’s correlation coefficient test was carried out to study correlations between parameters. A multiple correspondence analysis was performed in order to identify characteristics among individuals with MetS regarding their antioxidant–oxidant balance and metabolic management. A scree plot was used to choose the number of dimensions reliably representing the data, which enabled us to analyze the variables and present them as a Burt matrix. In addition, to assess the suitability of the selected markers for MetS diagnosis, a receiver operating characteristic (ROC) analysis was conducted. The Youden index was used to calculate the cutoff points that provided the highest possible accuracy of the parameter. To express the overall efficacy, the area under the ROC curve with 95% CI was examined. Values of *p* < 0.05 were considered statistically significant.

## 5. Conclusions

The presence of OS was observed in diabetic patients with MetS compared to both those without MetS (high OSI and low TAS values) and the control group (low levels of Zn, TAS, SOD, CAT and high of Cu/Zn, TOS, OSI). Furthermore, it was shown that the MetS− group of patients included those with well-balanced T1DM and additionally using either FGM or CGM systems. The study also identified the potential diagnostic usefulness of eGDR, OSI and HbA1c markers for the presence of MetS in young patients with T1DM.

## Figures and Tables

**Table 1:** Characteristics of the study cohort.

Parameter		MetS+ (n = 22)	MetS− (n = 81)	Control Group (n = 60)
Age (years)	Me (Q_1_–Q_3_)	13 (11–15)	14 (11–15)	15 (14–16)
Body height (cm)		167 (157–172)	163 (155–173)	167 (157–178)
Body weight (kg)		67 (58–71)	54 (45–66)	56 (47–67)
Age of diagnosis (years)		10 (7–11)	9 (6–11)	-
T1DM duration (years)		4 (1–6)	4 (1–7)	-
Gender (girls/boys)	%	68/32	47/53	40/60
Newly diagnosed (<2 years)		28	27	-
Type of insulin therapy (MDI/CSII)		27/73	44/56	-
Type of GMS (glucometer/FGM/CGM)		50/23/27	23/46/31	-

Values are expressed as median and interquartile range (Me (Q_1_–Q_3_) or percentage of respondents (%). Abbreviations: continuous glucose monitoring (CGM), continuous subcutaneous insulin infusion (CSII), flash glucose monitoring (FGM), glucose monitoring system (GMS), multiple daily injections (MDI), type 1 diabetes mellitus (T1DM).

**Table 2:** Comparison of cardiovascular, antioxidant defense, and oxidative stress parameters between MetS+ and MetS− patients, as well as the control group.

Parameter	MetS+ (n = 22)	MetS− (n = 81)	Control Group (n = 60)	*p*-Value (MetS+ vs. MetS−)	*p*-Value (MetS+ vs. Control)
Cardiovascular markers					
TC (mg/dL)	181 (169–197)	154 (128–175)	151 (127–172)	<0.001	<0.001
LDL (mg/dL)	103 (91–109)	83 (68–105)	87 (76–110)	<0.01	N/S
HDL (mg/dL)	45 (38–51)	57 (49–69)	57 (53–65)	<0.001	<0.001
TG (mg/dL)	122 (105–151)	66 (52–88)	59 (46–75)	<0.001	<0.001
SBP (mmHg)	122 (116–129)	114 (109–120)	118 (110–124)	<0.001	<0.05
DBP (mmHg)	79 (72–84)	71 (66–74)	71 (65–75)	<0.001	<0.001
BMI (kg/m^2^)	23.7 (20.8–25.2)	20.3 (18.4–21.9)	20.2 (18.6–21.8)	<0.001	<0.001
FGL (mg/dL)	-	-	99 (94–103)	-	-
HbA1c (%)	9.9 (7.9–11.2)	7.6 (6.6–9.1)	-	<0.01	-
eGDR (mg/kg/min)	4.4 (3.9–6.2)	7.5 (6.6–9.9)	-	<0.001	-
Antioxidant defense and oxidative stress markers					
Cu (mg/L)	0.914 (0.695–1.392)	0.863 (0.731–1.124)	0.935 (0.697–1.143)	N/S	N/S
Zn (mg/L)	0.875 (0.724–0.959)	0.896 (0.821–1.030)	0.976 (0.905–1.116)	N/S	<0.01
Cu/Zn ratio	1.113 (0.974–1.568)	0.995 (0.798–1.447)	0.978 (0.650–1.206)	N/S	<0.05
Se (µg/L)	57.0 (45.6–68.2)	61.3 (51.5–69.8)	61.4 (51.5–69.2)	N/S	N/S
TAS (mmol/L)	1.186 (1.040–1.336)	1.330 (1.201–1.540)	1.605 (1.477–1.766)	<0.05	<0.001
SOD (U/mL)	1.165 (0.960–1.716)	1.519 (1.154–2.100)	2.101 (1.699–2.298)	N/S	<0.001
CAT (n/mol/min)	49.1 (35.1–68.4)	42.1 (26.0–72.4)	57.7 (48.2–75.1)	N/S	<0.01
GPx (U/L)	1378 (908–2258)	1320 (728–2119)	1669 (987–3072)	N/S	N/S
TOS (μmol H_2_O_2_ equiv./L)	8.176 (7.456–10.295)	7.432 (5.892–9.189)	4.937 (3.920–5.857)	N/S	<0.001
OSI	0.666 (0.575–0.965)	0.533 (0.412–0.713)	0.284 (0.243–0.386)	<0.01	<0.001

Values are expressed as median and interquartile range (Me (Q_1_–Q_3_)). Statistically significant differences between the medians were detected by the Kruskal–Wallis ANOVA test with post-hoc analysis. Abbreviations: body mass index (BMI), catalase (CAT), copper (Cu), diastolic blood pressure (DBP), estimated glucose-disposal rate (eGDR), fasting glucose level (FGL), glutathione peroxidase (GPx), glycated hemoglobin (HbA1c), high-density lipoprotein (HDL), low-density lipoprotein (LDL), metabolic syndrome (MetS), non-significant (N/S), oxidative stress index (OSI), systolic blood pressure (SBP), selenium (Se), superoxide dismutase (SOD), total antioxidant status (TAS), total cholesterol (TC), triglycerides (TG), total oxidant status (TOS), zinc (Zn).

**Table 3:** Correlations between parameters among T1DM patients with MetS.

Parameter 1	Parameter 2	R	*p*-Value
**HbA1c**	TG	0.6	<0.01
	SBP	0.5	<0.05
	eGDR	−0.8	<0.001
	TAS	−0.5	<0.05
**eGDR**	TG	−0.5	<0.05
	SBP	−0.6	<0.01
**DBP**	eGDR	−0.7	<0.001
	BMI	0.6	<0.001
**SOD**	Zn	0.5	<0.05

Statistically significant correlations were detected using Spearman’s correlation coefficient. Repeated correlations between parameters were removed from the table. Abbreviations: body mass index (BMI), diastolic blood pressure (DBP), estimated glucose-disposal rate (eGDR), glycated hemoglobin (HbA1c), metabolic syndrome (MetS), systolic blood pressure (SBP), superoxide dismutase (SOD), triglycerides (TG), type 1 diabetes mellitus (T1DM), total antioxidant status (TAS), zinc (Zn).

**Table 4:** Assessment of oxidative stress and metabolic management parameters as possible indicators of the presence of MetS in patients with T1DM.

Parameter	TAS	TOS	OSI	HbA1c	eGDR
AUC (95% CI)	0.67 (0.53–0.81)	0.63 (0.51–0.75)	0.71 (0.59–0.82)	0.71 (0.60–0.83)	0.85 (0.74–0.95)
*p*-Value AUC	<0.01	<0.05	<0.001	<0.001	<0.001
Cutoff point	1.213	6.973	0.575	8.67	6.41
Sensitivity	59%	86%	77%	73%	82%
Specificity	74%	44%	57%	70%	78%
Youden index	0.332	0.308	0.341	0.431	0.596
+LR	0.259	0.556	0.432	0.296	0.222
−LR	0.409	0.136	0.227	0.273	0.182

Abbreviations: area under curve (AUC), confidence interval (CI), estimated glucose-disposal rate (eGDR), glycated hemoglobin (HbA1c), positive likelihood ratios (+LR), negative likelihood ratios (−LR), metabolic syndrome (MetS), oxidative stress index (OSI), type 1 diabetes mellitus (T1DM), total antioxidant status (TAS), total oxidant status (TOS).

**Table 5:** Values of the determined antioxidant defense and oxidative stress markers.

Parameter	Units	Wavelengths	Material
Cu	mg/L	324.8 nm	Serum
Se	µg/L	196 nm	Serum
Zn	mg/L	213.9 nm	Serum
TAS	mmol/L	600 nm	Serum
SOD	U/mL	450 nm	Serum
CAT	n/mol/min	540 nm	Serum
GPx	U/L whole blood	340 nm	Whole blood
TOS	μmol H_2_O_2_ equiv./L	560/800 nm	Serum

Abbreviations: catalase (CAT), copper (Cu), glutathione peroxidase (GPx), selenium (Se), superoxide dismutase (SOD), total antioxidant status (TAS), total oxidant status (TOS), zinc (Zn).

## Data Availability

The data presented in this study are available on request from the corresponding author.
